# MiR-195 regulates mitochondrial function by targeting mitofusin-2 in breast cancer cells

**DOI:** 10.1080/15476286.2019.1600999

**Published:** 2019-04-25

**Authors:** Paresh Kumar Purohit, Ruairidh Edwards, Kostas Tokatlidis, Neeru Saini

**Affiliations:** a Functional Genomics Unit, CSIR-Institute of Genomics and Integrative Biology, Delhi, India; b Academy of Scientific & Innovative Research, CSIR-Institute of Genomics and Integrative Biology, Delhi, India; c Institute of Molecular, Cell and Systems Biology, College of Medical, Veterinary and Life Sciences, University of Glasgow, Glasgow, UK

**Keywords:** MiR-195, Mitofusin-2, mitochondrial dysfunction, breast cancer cells

## Abstract

Mitochondrial dynamics is a highly dysregulated process in cancer. Apoptosis and mitochondrial fission are two concurrent events wherein increased mitochondrial fragmentation serves as a hallmark of apoptosis. We have shown earlier that miR-195 exerts pro-apoptotic effects in breast cancer cells. Herein, we have demonstrated miR-195 as a modulator of mitochondrial dynamics and function. Imaging experiments upon miR-195 treatment have shown that mitochondria undergo extensive fission. We validated mitofusin2 as a potential target of miR-195. This may provide a molecular explanation for the respiratory defects induced by miR-195 over-expression in breast cancer cells. Active, but not total, mitochondrial mass, was reduced with increasing levels of miR-195. We have further shown that miR-195 enhances mitochondrial SOD-2 expression but does not affect PINK1 levels in breast cancer cells. Collectively, we have revealed that miR-195 is a modulator of mitochondrial dynamics by targeting MFN2 thereby impairing mitochondrial function. Concomitantly, it enhances the scavenger of reactive oxygen species (SOD-2) to maintain moderate levels of oxidative stress. Our findings suggest a therapeutic potential of miR-195 in both ER-positive as well as ER-negative breast cancer cells.

## Introduction

Breast cancer is one of the leading causes of death in women worldwide. Treatment of breast cancer includes radiation, surgery and chemotherapy. The majority of breast tumours are hormone-dependent, and they account for about 70% of breast cancers. About 80% of breast cancers are ‘ER-positive’, i.e. the cancer cells grow in response to the hormone estrogen [1]. About 65% of these are also ‘PR-positive’ (Progesterone receptor).Tumours that are *ER*/*PR-positive* are much more likely to respond to hormone therapy than tumours that are *ER*/*PR*-negative.Triple negative breast cancer (TNBC) lacks all three receptors i.e.ER, PR, and Her2. The Cancer therapy that targets these receptors does not work well with TNBC tumour [2]. The development of drug resistance to chemotherapy is one of the major hurdles in treatment of breast cancer [3]. The existing therapeutic approaches are not sufficient to root-out breast cancer. Consequently, development of novel drugs that target tumours irrespective of their hormone receptor status has a potential to strengthen the fight against breast cancer.

**Figure 8.**
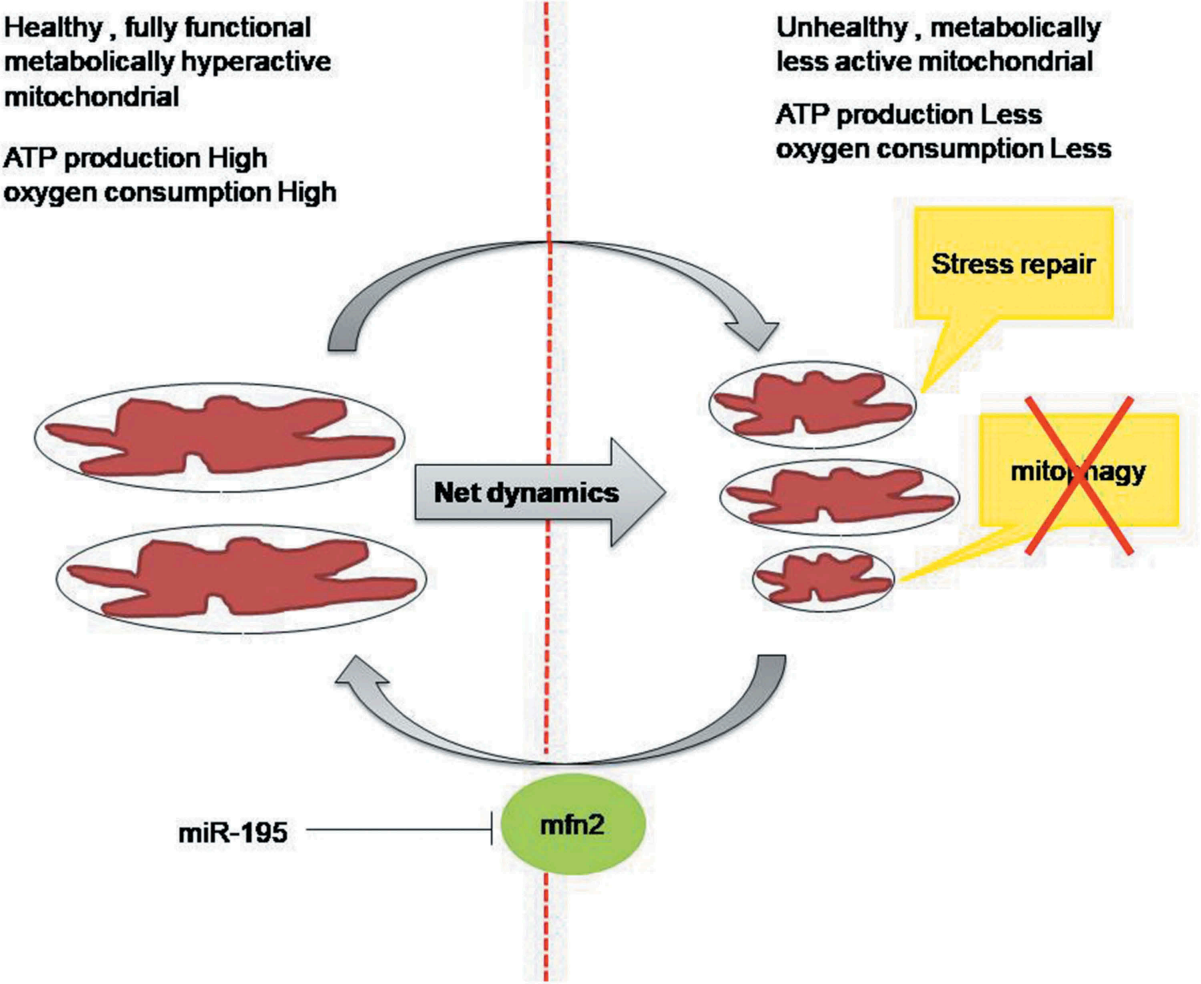
Schematic diagram showing miR-195 mediated mitochondrial functional impairment via MFN2. MiR-195 affects mitochondrial dynamics by blocking MFN2 mediated mitochondrial fusion. The fragmented mitochondria as a result of miR-195 over-expression are metabolically less active with repairable oxidative stress.

A growing number of studies have shown that microRNAs play key roles in the regulation of several cellular processes, whilst microRNA expression profiling has been associated with tumour development, progression and response to therapy [4,5].MiRNAs are 20–25 nucleotide long sequences of non-coding RNA that regulate gene expression post-transcriptionally. MiRNAs mimics and antagomiR have been widely used to normalize miRNA expression and hence gene expression networks in disease conditions [6]. The ability of miRNA to restore gene expression to physiologically normal level renders microRNA-based strategies a potential therapeutic approach.

In several studies, hsa-miR-195 has been reported to be differentially expressed in normal and breast cancer tissues [7–10]. A decrease in hsa-miR-195 expression in cancer tissue is due to increased methylation on the CpG Island of the miR-195 promoter [11,12]. Further, increased circulatory level of hsa-miR-195 in breast cancer patients and decline in circulatory miR-195 post-operation make hsa-miR-195 a potential biomarker for breast cancer [13–15]. In previous studies, we have unveiled a pro-apoptotic role of miR-195 in breast cancer and we have demonstrated that miR-195 downregulates Bcl2 by direct binding to its 3ʹUTR Sequence [16]. In another study, we have further shown the anti-proliferative, non-invasive and anti-metastatic role of hsa-miR-195 in breast cancer cells [17]. These findings suggest hsa-miR-195 has strong anticancer properties with a promising therapeutic potential in breast cancer. Besides its pro-apoptotic role, hsa-miR-195 has also been shown to depolarize the mitochondrial inner membrane and to increase the calcium concentration in mitochondria [17]. Despite all previous studies, it remains unclear how miR-195 affects mitochondrial function. During our *in-silico* analysis we observed that mitofusin-2(MFN2) a predicted target of hsa-miR-195. Further analyzing our illumina microarray data, we found a differential expression of MFN2 upon over-expression of miR-195 in breast cancer cells. Herein, we have validated mitofusin-2 as a direct target of miR-195 in breast cancer cell lines. Over-expression of miR-195 in breast cancer cell induces mitochondrial fragmentation and diminishes the ability for mitochondria to consume oxygen. The observed functional impairment of mitochondria induced by miR-195 is independent of the ER (Estrogen receptor) status of cells.

## Results

### MiR-195 down regulates MFN2 in breast cancer cell lines

**Figure 1.**
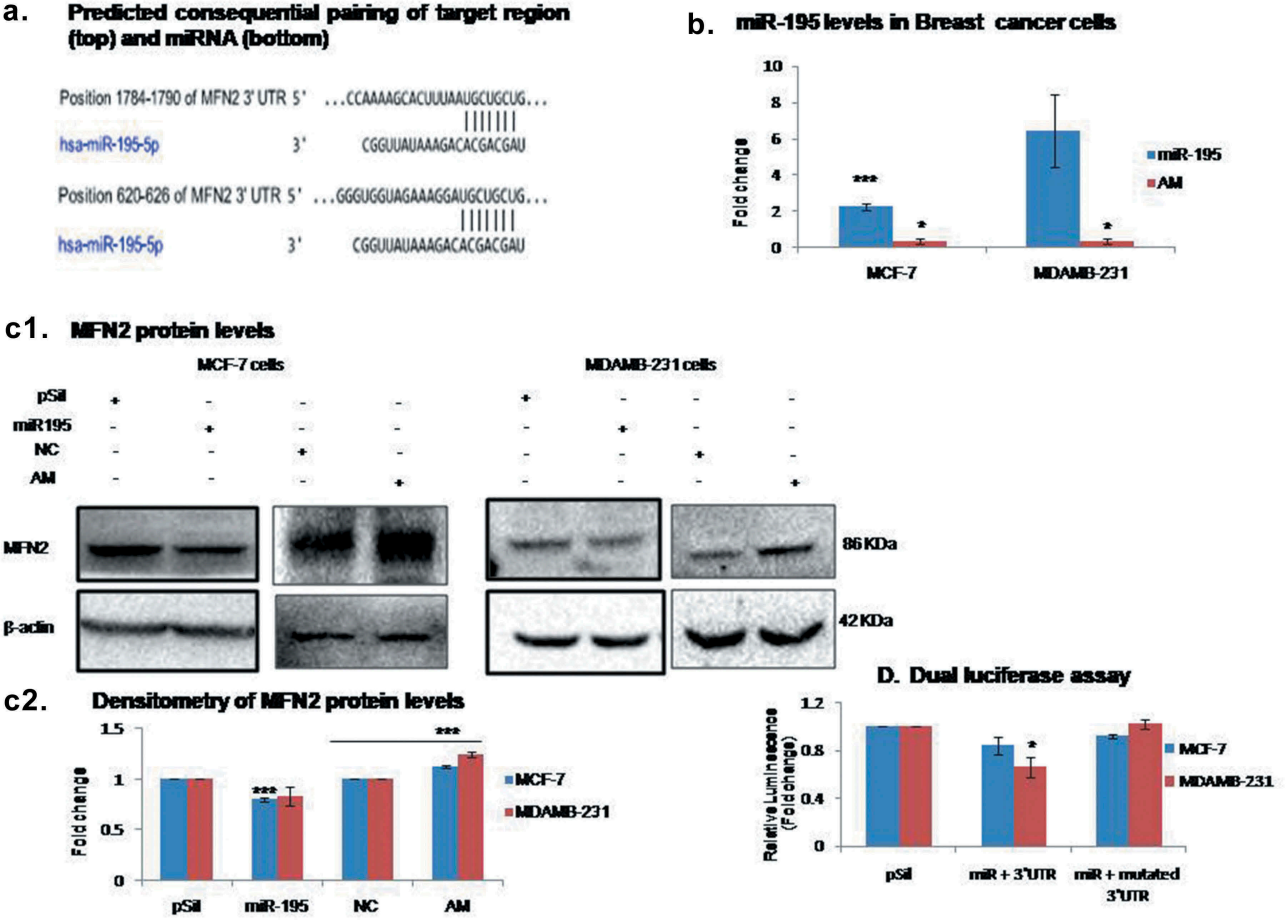
MiR-195 downregulates mitofusin-2 (mfn-2) by directly binding to its 3ʹUTR sequence. (a) Predicted binding sites of miR-195 on 3ʹUTR sequence of MFN2 m-RNA **(b)** MiR-195 over-expressed using p195 plasmid construct (miR-195) and knockdown using antimiR-195 (AM) in MCF-7 and MDAMB231 cells **(c1)**MiR-195 over-expression depleted MFN2 protein levels while knockdown of miR-195 enhances MFN2 protein levels in MCF-7 and MDA-MB-231cells **(c2)**Mean fold change in MFN2 level ±SE for n = 3 was plotted **(d) **Over-expression of miR-195 diminishes relative luminescence in breast cancer cells, Representative plot of luciferase assay, mean fold change ±SE for n = 3 was plotted,*: *p*< 0.05, ***: *p*< 0.001. (pSil, miR-195, NC,AM represents treatment of control plasmid, miR-195 plasmid, negative control and antimiR-195, respectively)

We have previously shown that miR-195 affects mitochondrial function by means of depolarization of the inner membrane and disturbing calcium homeostasis within the organelle [17]. However, the exact mechanism by which these processes are controlled was not investigated. *In-silico* analysis using target scan revealed a miR-195 binding site in the 3ʹUTR of MFN2 (Figure 1(A)). Mitofusin-2 is a crucial protein known to play a role in controlling mitochondrial dynamics and maintenance of mitochondrial calcium homeostasis. The miR-195 was upregulated using pSilencermiR-195 (miR) cloned vector whereas downregulated using antimiR-195 (AM)(Figure 1(B)). As shown in Figure 1(C), western blot analysis revealed significant downregulation (p-value<0.0005) of MFN2 upon over-expression of miR-195 in MCF-7 and MDA-MB-231 cells. While, MFN2 levels were significantly increased (p-value<0.0005) when miR-195 expression was reduced in MCF-7 and MDA-MB-231 cell lines. The dual luciferase assay was performed to check direct binding of miR-195 to 3ʹUTR sequence of MFN2. The luciferase activity was reduced by 0.4 fold (p-value<0.05) upon up-regulation of miR-195 in MDA-MB-231 cells whereas 0.16 fold decrease in luciferase activity was observed in MCF-7 cells (Figure D), The luciferase activity was restored back to normal when the miR-195 binding site was deleted from the luciferase assay plasmid (pSichek) further confirming direct binding of miR-195 to the 3ʹUTR sequence of MFN2.

### MiR-195 affects mitochondrial morphology

**Figure 2.**
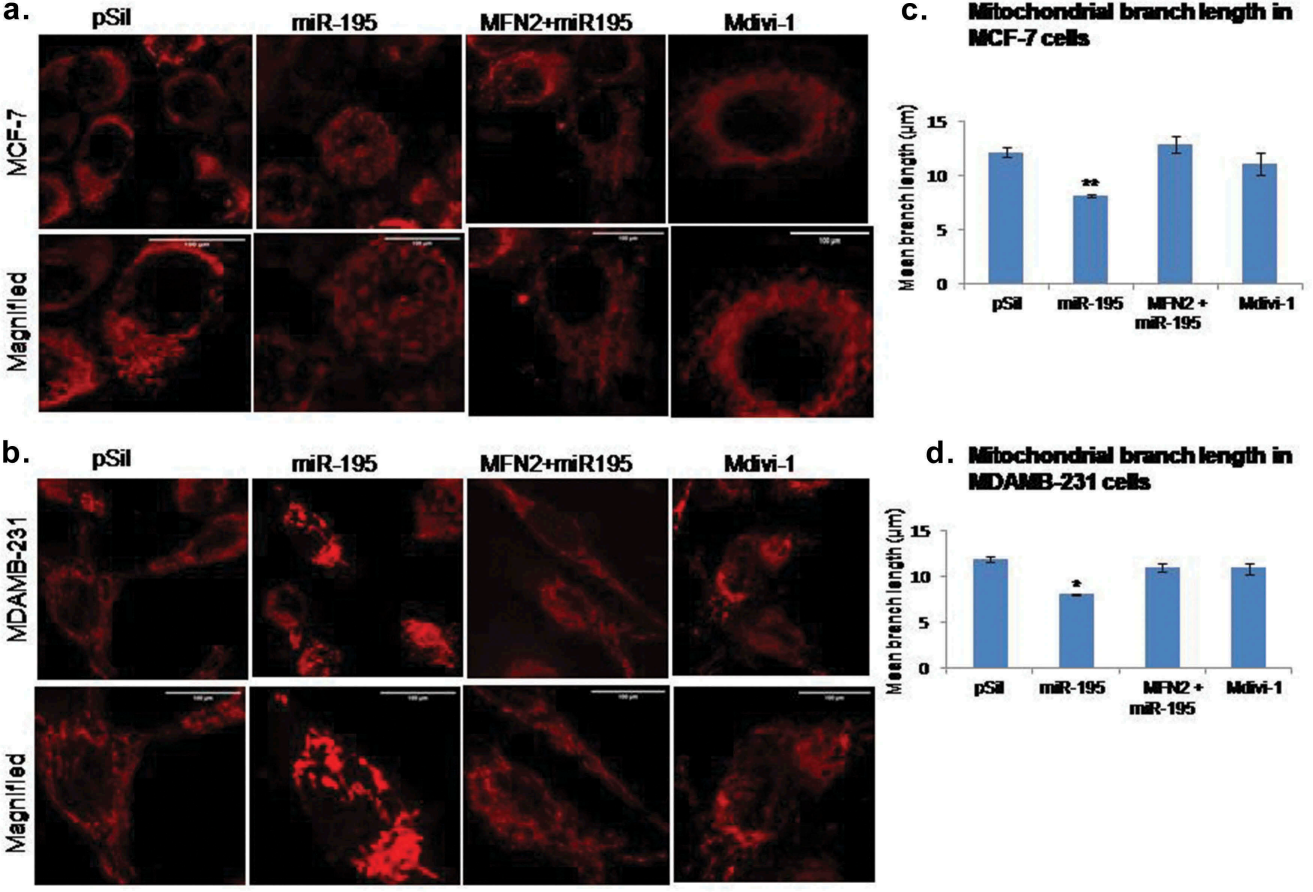
Mitofusin-2 down-regulation by miR-195 changes mitochondrial morphology and promote mitochondrial fission. Mitochondria become rounded, small and fragmented upon up-regulation of miR-195 and gets tubular in shape upon treatment of MFN2 along with miR-195 and Mdivi-1 treatment in **(a)** MCF-7 cells and **(b)** MDAMB-231 cells. Bar: 100 µm. The mean branch length of mitochondria reduces upon miR-195 treatment and restored back upon treatment of MFN2 along with miR-195 and Mdivi-1 treatment in **(c)**MCF-7 and **(d)**MDAMB-231, mean branch length ±SE for n = 3 was plotted, *: *p*< 0.05, ***: *p*< 0.001.

Mitochondrial morphology is a critical factor that determines its activity. MFN2 has been shown to affect mitochondrial dynamics by promoting fusion events and hence the mitochondrial morphology. Mitotracker Red CMXRos has been used to check morphology of the organelle. We observed increase fragmentation of mitochondria when we over-expressed miR-195 in MCF-7 and MDA-MB-231 cell lines (Figure 2(A,B)). The highly networked tubular mitochondria in control (pSilencer treated) cells tend to become rounded, small an fragmented in shape upon over-expression of miR-195 in both cell lines, the mitochondrial shape was restored back to elongated tubular even upon up-regulation of miR-195 when cells were treated with MFN2 that does not contain the 3ʹUTR sequence (pMfn2). This confirms that the 3ʹUTR sequence of MFN2 is requisite for miR-195 to enhance mitochondrial fission. The mitochondrial division inhibitor Mdivi-1 was used as a positive control of fusion events and hence tubular shape of organelle was observed upon treatment of Mdivi-1 in both breast cancer cell lines (Figure 2(A,B)).The mean branch length of mitochondria was reduced by 0.4 fold upon up-regulation of miR-195 in MCF-7 (p-value<0.05) and MDAMB-231 (p-value<0.01) cells, respectively (Figure 2(C,D)). The mean branch length was restored back like control (pSilencer treated) when cells were treated with pMfn2 along with miR-195. Mdivi-1 treatment also enhanced mitochondrial branch length in comparison to miR-195 treatment in both breast cancer cells (Figure 2(C,D)).

### MiR-195 promotes mitochondrial fission by blocking fusion events

**Figure 3.**
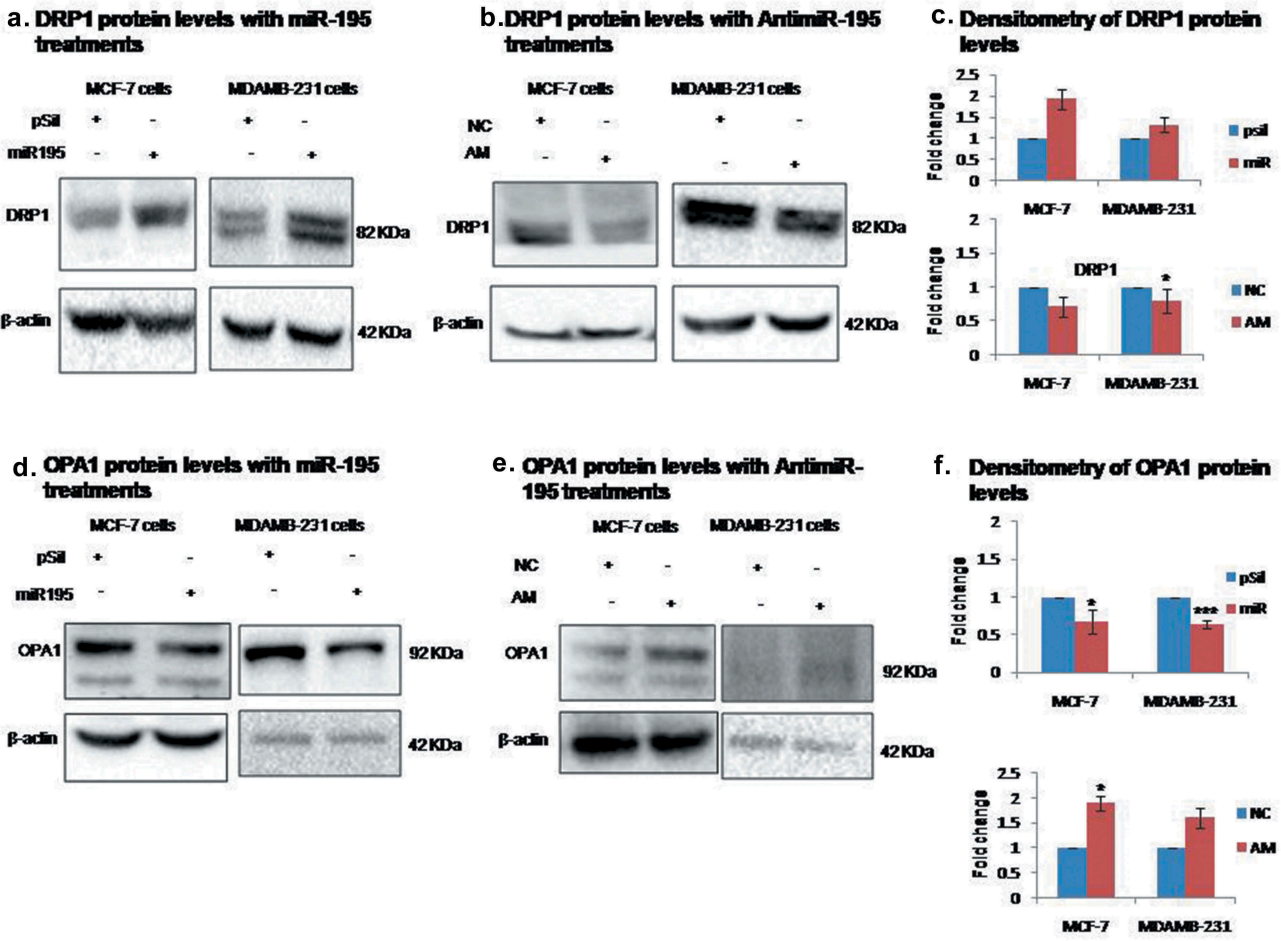
MiR-195 affects mitochondrial dynamics by blocking fusion. **(a)** Dynamin-related protein (DRP1) gets upregulated upon over-expression of miR-195 in MCF-7 cells (right panel) and MDA-MB-231 cells (left panel) **(b)** Knockdown of miR-195 using antimiR-195 (AM) reduces DRP1 protein levels in MCF-7 cells (right panel) and MDA-MB-231 cells (left panel) **(d)** Mitochondrial inner membrane GTPase (OPA1) gets down-regulated upon over-expression of miR-195 in MCF-7 cells (right panel) and MDA-MB-231 cells (left panel) **(e)** Depletion of miR-195 using AM enhances OPA1 levels in MCF-7 cells (right panel) and MDA-MB-231 cells (left panel) **(c, f)** Mean fold change ±SE for n = 3 was plotted, *: *p*< 0.05, ***: *p*< 0.001. (pSil, miR, NC, AM represents treatment of control plasmid, miR-195 plasmid, negative control and antimiR-195, respectively)

Mitochondrial morphology is critical for its function and it is regulated by mitochondrial dynamics. Apart from mitofusins there are other proteins whose levels change markedly during mitochondrial fusion and fission events. Here we found that up-regulation of miR-195 affects the levels of both DRP1 (dynamin-related protein) and OPA1. Specifically, in MCF-7 cells, DRP1 increases 1.9-fold (Figure 3(A) first panel) whilst OPA1 decreases 0.32-fold (Figure 3(D) first panel). In MDAMB-231 cells 1.32-fold increase in DRP1 level was observed (Figure 3(A) second panel) whereas a 0.36-fold decrease in the OPA1 level (Figure 3(D) second panel)

Conversely, knockdown of miR-195 has different effects. In MCF-7 cells DRP1 decreases 0.3-fold (p-value<0.05) (Figure 3(B) first panel) and OPA1 increases 1.9-fold (Figure 3(E) first panel). In MDAMB-231 cells DRP1 decreases 0.2-fold (Figure 3(B) second panel) whilst OPA1 increases 1.60-fold (Figure 3(E) second panel) The antimiR-195 treatment in both cell lines shows exactly opposite effects to that of miR-195. The enhanced DRP1 expression and reduced OPA1 protein level is a sign of miR-195 induced increase in mitochondrial fission events in breast cancer cell lines.

### MiR-195 diminishes active mitochondrial mass but does not promote degradation of mitochondria through mitophagy

**Figure 4.**
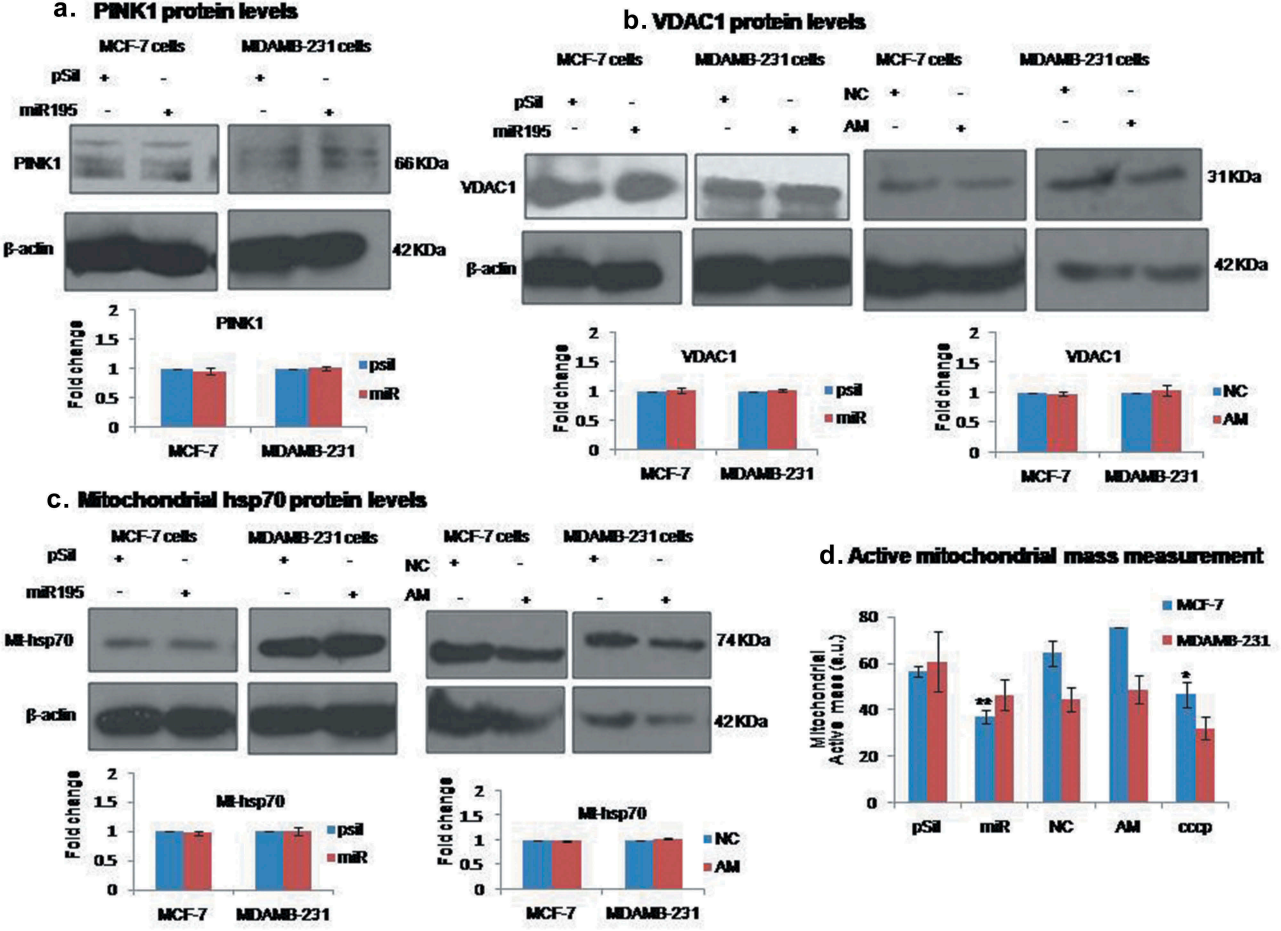
MiR-195 diminishes Active mitochondrial mass but not the total mass of mitochondria. **(a)** Mitophagy marker PINK1 levels were not significantly changed with differential expression of miR-195 in MCF-7 (right panel) and MDA-MB-231 cells (left panel). The alteration in miR-195 level does not affect the protein level of **(b)** VDAC1 (mitophagy and mitochondrial mass marker) as well **(c)** mt-hsp70 (mitochondrial mass marker) in both breast cancer cell lines. Mean fold change ±SE for n = 3 was plotted **(d)** Active mass of mitochondria was reduced upon miR-195 over-expression whereas active mass got resumed to normal levels with depletion of miR-195 in MCF-7 and MDA-MB-231 cells, the positive control CCCP treated cells have shown lesser mitochondrial active mass, plot is representation of fold change in mean fluorescence intensity ±SE, *: *p*< 0.05, ***: *p*< 0.001. (pSil, miR, NC, AM represents treatment of control plasmid, miR-195 plasmid, negative control and antimiR-195, respectively)

Mitophagy and mitochondrial dynamics are two inter-connected processes needed to maintain fully functional mitochondria by targeting defective and damaged mitochondria to a dedicated degradation process. Mitophagy is not the only process by which a healthy pool of mitochondria is maintained since damaged mitochondria can also undergo a repair pathway or they can fuse with completely healthy mitochondria and compensate the damage. As fusion events are blocked by miR-195 through the targeting of MFN2, therefore, fusion of damaged mitochondria with healthy ones is no longer an option for mitochondria to overcome the damaging oxidative stress. We have therefore checked for mitochondrial mass and mitophagy. The VDAC1 and mtHSP70 proteins are used as mitochondrial mass markers whilst PINK1 is used as a mitophagy marker. Upon over-expression of miR-195 or knockdown of miR-195, none of these markers (Figure 4(A) for PINK1, 4B for VDAC1, 4C mtHSP70 protein) have shown any significant difference compared to the vehicle control in MCF-7 and MDA-MB-231 cell lines. These observations clearly suggest that miR-195 does not promote degradation through mitophagy of damaged mitochondria and hence total mitochondria mass does not change. We further probed for the functionally active mitochondria pool using Mitotracker Red CMXRos (which is highly dependent on a functional mitochondrial transmembrane potential). We observed a decrease of active mass of mitochondria upon miR-195 treatment in MCF-7 and MDA-MB-231 cell lines. On the other hand, the mass of active mitochondria increased upon down-regulation of miR-195 in both cell lines. CCCP treatment was used as a positive control. Taken together, these observations suggest that miR-195-mediated mitochondrial membrane depolarization is not severe enough to induce mitophagy, but it is sufficient to diminish mitochondrial activity and hence the pool of active mitochondria.

### MiR-195 induces a defect in mitochondrial respiration

**Figure 5.**
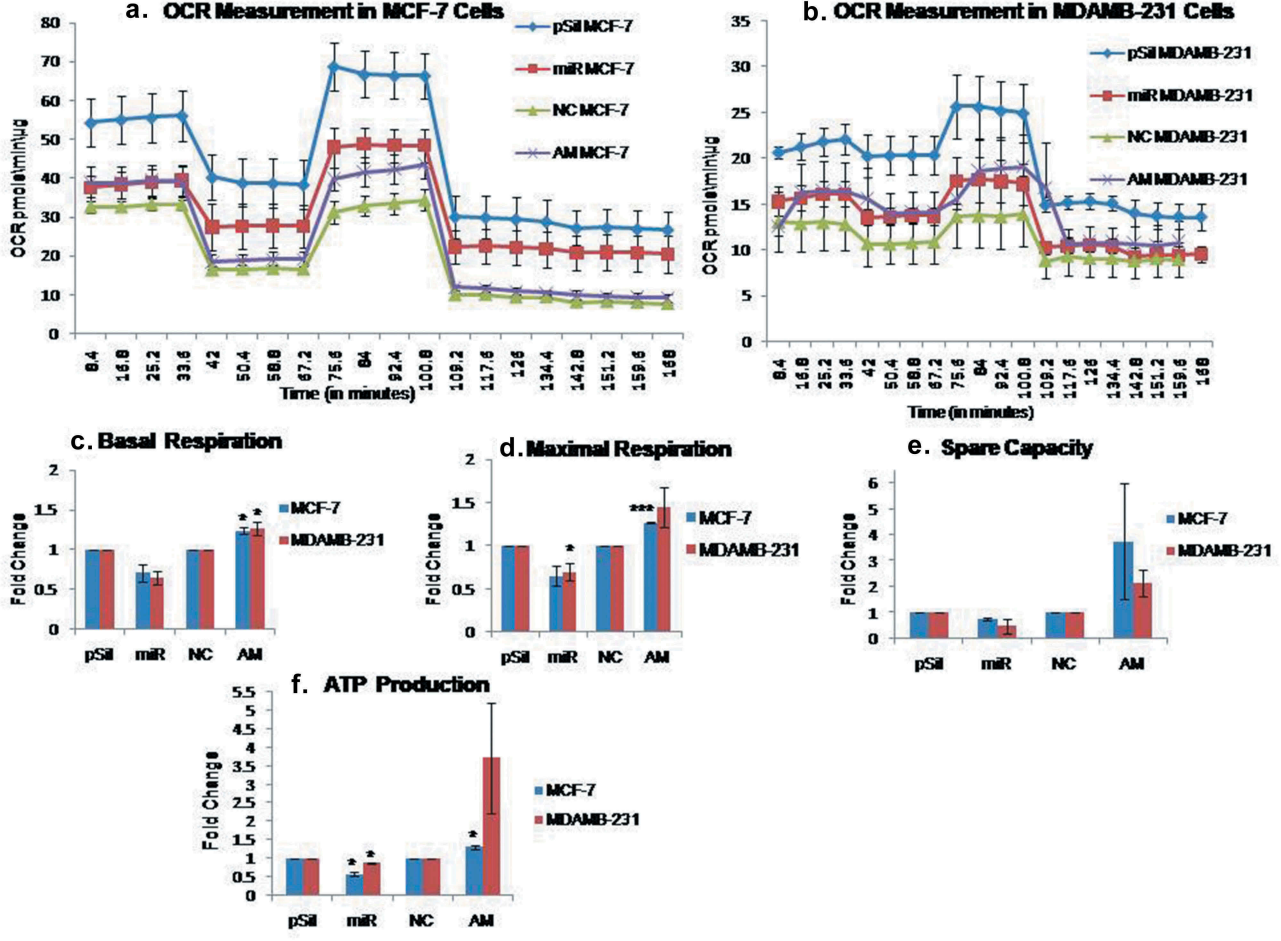
MiR-195 reduces oxygen consumption rate via MFN2 down-regulation. Elevated levels of miR-195 reduces OCR whereas declining miR-195 levels augments OCR in **(a)** MCF-7 cells and **(b)** MDA-MB-231 cells, normalized OCR per min per microgram of protein ±SE from n = 3 was plotted. The respiratory parameters such as **(c)** Basal respiration, **(d)** Maximal respiration, **(e)** Spare respiratory capacity of mitochondria and **(f)** ATP production was derived from OCR and mean fold change was plotted ±SE, *: *p*< 0.05, ***: *p*< 0.001. (pSil, miR, NC, AM represents treatment of control plasmid, miR-195 plasmid, negative control and antimiR-195, respectively)

To further validate the effect of miR-195 on aerobic respiration, the oxygen consumption rates of mitochondria (OCR) measured directly with a SeaHorse-XF24 flux analyser. The over-expression of miR-195 shows a respiratory defect as OCR is reduced, whilst down-regulation of mir-195 resulted in an increase in OCR in both the MCF-7 and the MDA-MB-231 cell lines. The OCR was used to further calculate other respiratory parameters such as basal respiration, maximal respiration, ATP production and spare respiratory capacity of mitochondria. miR-195 appears to attenuate basal respiration by 0.3 fold in MCF-7 and 0.35 fold in MDA-MB-231 cell lines whereas antimiR-195 treatment increases basal respiration by 1.24 fold and 1.27 fold in MCF-7 and MDA-MB-231 cell lines, respectively (Figure 5(C)), The maximal respiration also decreases by 0.35 fold and 0.3 fold in MCF-7 and MDA-MB-231 cell lines respectively, on contrary down-regulation of miR-195 enhances maximal respiration by 1.27 fold in MCF-7 and 1.47 fold in MDA-MB-231 cell lines (Figure 5(D)). As shown in Figure 5(E), miR-195 over-expression diminishes spare respiratory capacity by 0.33 fold and 0.51 fold in MCF-7 and MDA-MB-231 cell line respectively, on the other hand antimiR-195 increases spare capacity of mitochondria by 3.75 fold in MCF-7 and 2.16 fold in MDA-MB-231 cell lines. The OCR derived ATP production also decreases by 0.42 fold and 0.22 fold in MCF-7 and MDA-MB-231 cell lines, respectively, upon up-regulation of miR-195, conversely reduction in miR-195 level by antimiR-195 augments ATP production by 1.3 fold in MCF-7 and 3.72 fold in MDA-MB-231 cell line (Figure 5(F)).

### Oxidative stress is augmented by miR-195

**Figure 6.**
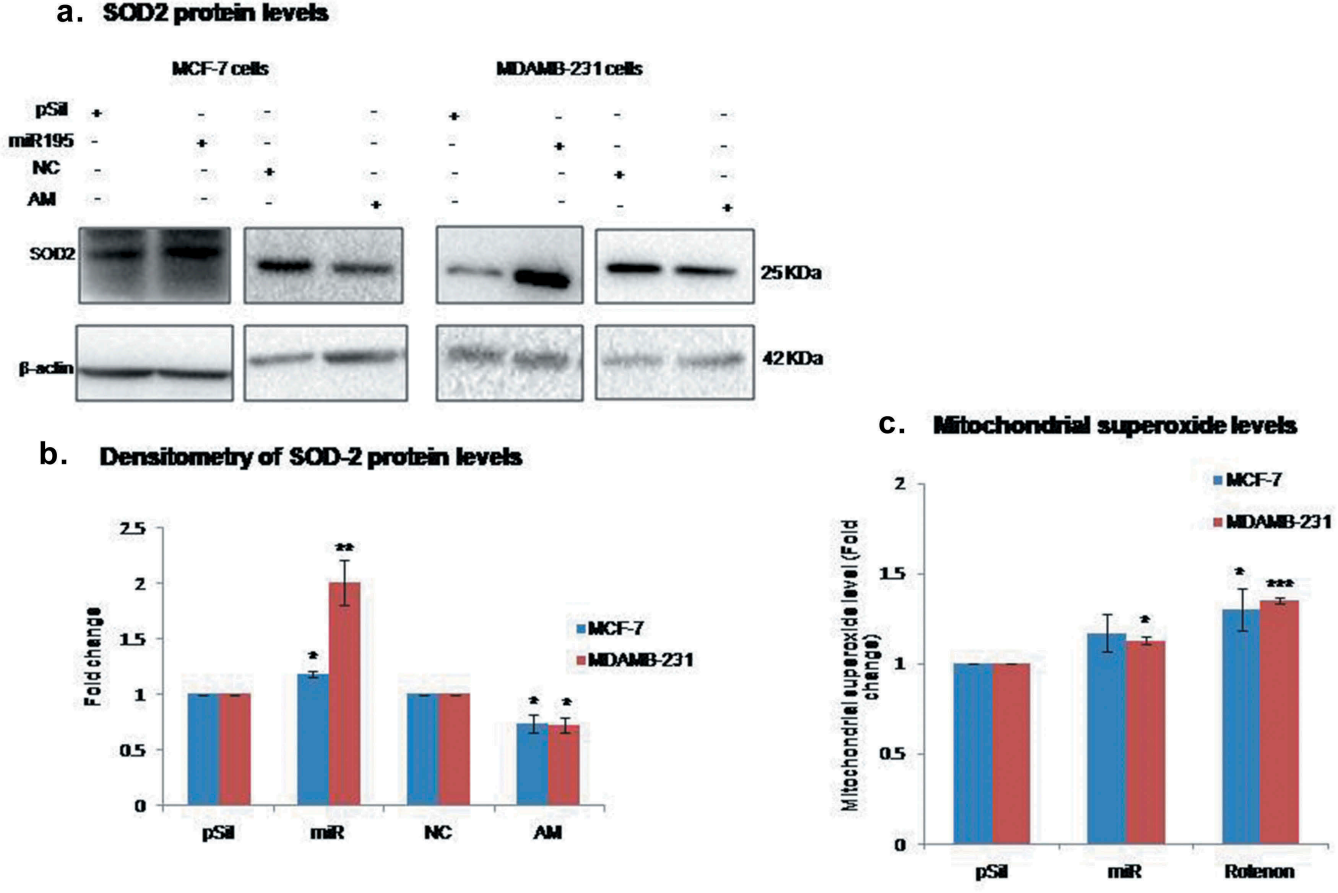
Moderate levels of mitochondrial oxidative stress are maintained by miR-195. **(a)** Mitochondrial superoxide scavenger (mn-SOD2) elevates with miR-195 up-regulation whereas mn-SOD2 levels declined upon depletion of miR-195 in MCF-7 and MDA-MB-231 cells. **(b)** Mean fold change ±SE for n = 3 was plotted. **(c)** Mitochondrial superoxide level was augmented mildly upon over-expression of miR-195 in both breast cancer cells, relative fold change in mean fluorescence ±SE was plotted from n = 3 for MDA-MB-231 and n = 4 for MCF-7, *: *p*< 0.05, ***: *p*< 0.001. (pSil, miR, NC,AM represents treatment of control plasmid miR-195 plasmid, negative control and antimiR-195, respectively)

The mitochondria are a prime source of energy production in cells, wherein, they utilizes the proton gradient across the membrane to generate ATP and convert molecular oxygen into water as a byproduct. The process of oxidative phosphorylation and electron transfer constantly takes place in inner mitochondrial membrane of metabolically active cells. The activity and proper assembly of electron transport chain complexes plays a central role in the electron flow and generation of the proton gradient across the membrane. Any defect in the ETC components leads to leakage of electrons to the mitochondrial matrix and that in turn converts molecular oxygen to superoxide, a damaging ROS. To check the effect of miR-195 on oxidative stress, we have probed for mitochondrial superoxide using MitoSox Red, a fluorescent probe that is rapidly oxidized by superoxides in mitochondria and produces red fluorescence. The over-expression of miR-195 enhanced superoxide levels 1.2- fold in MCF-7 cells and 1.13-fold in MDA-MB-231 cells (Figure 6(C)). This increase in mitochondrial ROS suggests the involvement of ETC in the effect exerted by miR-195 on mitochondrial dynamics. To further examine the effects on ROS, we checked the expression of MnSOD2 as it is the key scavenger of superoxide in the matrix of mitochondria. Interestingly, the level of MnSOD2 increased 1.2-fold (p-value<0.05) in MCF-7 cells and 2.0 fold (p-value<0.01) in MDA-MB-231 upon over-expression of miR-195 (Figure 6(A,B)). Conversely, the MnSOD2 level decreased 0.3-fold (p-value<0.05) in both MCF-7 and MDA-MB-231 cells when miR-195 was down-regulated (Figure 6(A,B)). This additional evidence supports the role of miR-195 in maintaining the mitochondrial superoxide concentration at non-damaging levels by up-regulating its main scavenger protein (MnSOD2) and hence bypassing the mitophagy process.

### Apoptosis induced by MiR-195 is independent of MFN-2 mediated mitochondrial dysfunction

**Figure 7.**
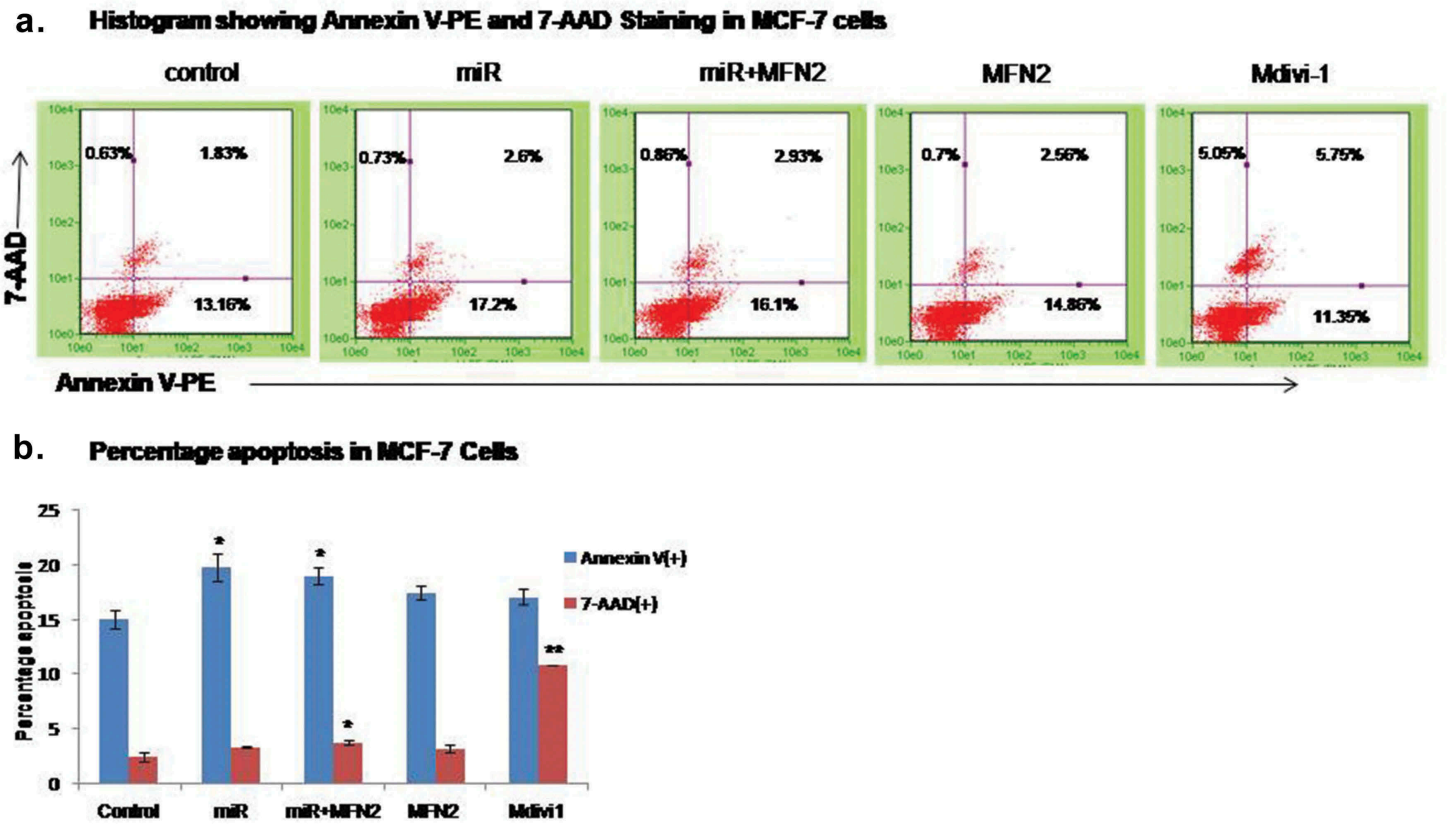
Apoptosis induced by MiR-195 is independent of MFN-2 mediated mitochondrial dysfunction. **(a)** Histogram of Annexin V-PE and 7-AAD binding upon treatment of miR-195, MFN2 along with miR-195, MFN2 and Mdivi-1 in MCF-7 cells. **(b)** Plots are a representation of relative percentage apoptosis ±SE from n = 3 in MCF-7, *: *p*< 0.05, ***: *p*< 0.001.

Mitochondrial superoxide generation is often associated with increased cell death [18]. To further investigate the effect of miR-195 mediated mitochondrial dysfunction on apoptosis, an Annexin-V apoptosis assay was performed. The over-expression of miR-195 enhanced apoptosis (Annexin-V positive cells) by 1.33 fold (p-value<0.05) in MCF-7 cells (Figure 7(B)) whereas in MDAMB-231 cells an insignificant increase in apoptosis was observed (supplementary Figure 1B). There was also a significant increase in apoptosis (p-value<0.05) when both the cell lines were treated with pMfn2 (construct without UTR) along with miR-195 as compared to their respective controls (Figure 7(B), supp. Figure 1B). Our findings suggest that the pro-apoptotic effects exerted by miR-195 did not get reversed upon restoring MFN2 levels in both breast cancer cell lines. Hence, the induction of apoptosis by miR-195 is independent of MFN2 mediated mitochondrial dysfunction. Furthermore, the blocking of mitochondrial fission by chemical inhibitor Mdivi-1 also caused significantly increased apoptosis (p-value<0.01) in both cell lines (Figure 7(B),supp. Figure 1B). Taken together these findings indicate that any disturbance in mitochondrial dynamics and hence mitochondrial quality control leads to enhanced apoptosis.

## Discussion

Healthy mitochondria play a critical role in the determination of cell fate and the maintenance of cellular homeostasis. Mitochondrial dysfunction is a pathological hallmark for different diseases and stress-induced mitochondrial dysfunction is known to activate the intrinsic pathway of apoptosis [19–22]. The dynamic nature of mitochondria ensures their quality, whilst unhealthy mitochondria with irreparable damage undergo a specific degradation process called mitophagy [23–27]. A healthy pool of mitochondria is maintained by a series of fusion-fission events. Mitofusin1 and Mitofusin2 facilitate fusion of the outer mitochondrial membrane [28–30], while the inner mitochondrial membrane gets fused by the inner membrane GTPase protein OPA1 [31–33]. Mitochondrial fission is triggered upon localization of the DRP1 and FIS1 proteins on the scissor site [34–37]. In conclusion, mitochondrial dynamics and mitochondrial morphology are highly linked processes that play a major role in mitochondrial quality control to maintain cellular homeostasis.

Previously we demonstrated that hsa-miR-195 targets Bcl-2, it causes mitochondrial dysfunction, it induces apoptosis and augments the effect of etoposide in breast cancer cells. We further demonstrated that miR-195 also targets genes involved in de novo lipogenesis, it inhibits cell proliferation, migration, and invasion by targeting FASN, HMGCR, ACACA and CYP27B1 thereby potentially opening new avenues for the treatment of breast cancer. However, the underlying mechanism by which miR-195 causes mitochondrial dysfunction remains unexplored. MicroRNAs that affect mitochondrial function have been explored and studied in various disease contexts [38–40]. Wherein miR-668 has been shown to affect mitochondrial dynamics during ischemic acute kidney injury [41]. Similarly, Juan Ji et.al. has also reported that miR-141-3p targets PTEN to induce mitochondrial dysfunction in high fat diet induced obesity [42].

Herein, we first checked whether miR-195 can regulate mitochondrial shape and function. Our current findings revealed that miR-195 modulates mitochondrial shape and enhances fragmentation of mitochondria in breast cancer cells (Figure 2). We also identified MFN2 to be a direct target of miR-195 in apoptotic breast cancer cell lines. The miR-195 has been previously reported to target MFN2 in Alzheimer's disease mice [43] but the correlation of miR-195 and MFN2 and its implication on mitochondrial dynamics has not been explored before in breast cancer. The expression of MFN2 has been shown by other groups to be implicated in various cancers [44]. The over-expression of MFN2 has been observed to enhance tumorigenesis while inhibition of MFN2 has been revealed to exert anti-cancer effects in human gastric cancer [45]. Further, MFN2 has been shown to affect mitochondrial dynamics by promoting fusion events. In an independent study, Leboucher and colleagues reported that the phosphorylation and proteosomal degradation of MFN2 under stress condition facilitates mitochondrial fission and apoptosis [46]. On the contrary, Li Ma et al. have shown that MFN2 induces apoptosis via PI3K/Akt signaling in breast cancer cells [47]. In addition to this, Xu K et al. further reported that MFN2 suppresses cancer progression by inhibition of the mTORC2/Akt signalling pathway [48]. Furthermore, 17β-estradiol has been observed to enhance proliferation and migration of MCF-7 cells by down-regulating mfn2 [49]. Therefore, the role of MFN2 in apoptotic pathways is contextual.

We further demonstrated that miR-195 reduces the efficiency of mitochondria to consume oxygen. The observed differential levels of OPA1 and DRP1 upon alteration of miR-195 expression further suggested an indirect involvement of both proteins in modulation of mitochondrial dynamics by miR-195. Next, we were curious to know the fate of fragmented mitochondria in apoptotic breast cancer cells upon up-regulation of miR-195. Mitochondrial fission and increased oxidative stress is often known to decrease mitochondrial mass by committing damaged mitochondria to mitophagy. However, to our surprise, we did not observe any change in mitochondrial mass and hence no mitophagy but there was a decrease in the mass of active mitochondria. Taken together, these observations suggest that miR-195 affects mitochondrial functions and enhances oxidative stress but the stress is not sufficient to trigger mitophagy (Figure 8). It seems that miR-195 induces moderate oxidative stress to compromise mitochondrial activity and that is the reason behind a reduced mass of active mitochondria (Figure 8).

Further, to check how the moderate level of stress is being maintained by miR-195, we analyzed the mitochondrial MnSOD2 and observed significant upregulation of superoxide scavenging in mitochondria under the influence of miR-195. This illustrates a very subtle effect of miR-195 that enhances oxidative stress on the one hand by affecting mitochondrial dynamics, whilst it does not allow excess ROS accumulation by enhancing the level of the scavenger (MnSOD2) (Figure 8).

Recently it has been shown that the mitochondrial fission machinery plays a critical role in mitochondrial fragmentation during apoptosis [50]. Herein, we have measured apoptotic cell death induced by miR-195. The restoration of MFN2 level with deleted 3ʹUTR sequence (pMfn2) did not decrease miR-195 induced apoptosis thereby suggesting that the pro-apoptotic role of miR-195 is MFN2-independent in breast cancer cells. The induction of apoptosis by down-regulation BCL2 [16] and mitochondrial fragmentation via reduction of MFN2 levels by miR-195 seem to be two parallel events. Wherein, mitochondrial fragmentation by miR-195 during apoptosis is attained by MFN-2 down-regulation.

Our results revealed miR-195 mediated potential respiratory defects in MCF-7 and MDA-MB-231 cell lines that characterize miR-195 as a modulator of mitochondrial respiration. The current study gives detailed mechanistic insight into the mitochondrial functional impairment by miR-195 in apoptotic breast cancer cell lines. The results presented here expand our previous finding that miR-195 mediates differential expression of components of the mitochondrial electron transfer chain in breast cancer cell lines [17]. The present study provides a much needed framework to gain further insight into the role of miR-195 as a putative potential therapeutic molecule not just in cancer but various metabolic as well as neurodegenerative diseases where mitochondrial function is compromised.

## Materials and methods

### Cell culture

Human breast adeno carcinoma cell lines MCF-7 and MDA-MB-231 were procured from the National Centre for Cell Sciences (NCCS), Pune, India and cultured using DMEM containing 10% (v/v) fetal calf serum, 100 Units/ml penicillin, 100 μg/ml streptomycin, 0.25 μg/ml amphotericin at 37°C in a humidified atmosphere at 5% CO2.

### MiR-195 over-expression and knockdown

Over-expression of miR-195 was achieved using p195 (237 bp sequence containing premiR-195 cloned in psilencer 4.1 vector purchased from Ambion, Austin, TX, USA) [16] and pSilencer 4.1 empty vector was used as a control. The miR-195 knockdown was done using AntimiR-195 oligos (AM17000) and AntimiR miRNA inhibitor negative control (AM17010). Both oligos were purchased from Ambion, Austin, TX, USA. Lipofectamine 2000 and Lipofectamine LTX-Plus (Invitrogen, CA, USA) were used for transfection according to manufacturer’s protocol. Cells were trypsinized and harvested 24 hours post-transfection. The MFN2 plasmid without the 3ʹUTR sequence (pMfn2) was purchased from addgene (plasmid number 28010) [51]. We are working with miR-195-5p in this manuscript.

### Mitochondrial morphology

The mitochondrial shape was visualized using the mitochondria-specific probe mitotracker CMXRos [52]. CMXRos is a fluorescent dye that is lipophilic and cationic in nature. CMXRos binds to the negatively charged matrix side of the mitochondrial inner membrane. Cells were stained with 200 nM CMXRos for 15 min at 37 ⁰C post miR-195 treatments in a cell culture incubator. The MFN2 plasmid without the 3ʹUTR sequence (pMfn2) was purchased from addgene (plasmid number 28010) [51] and used for over expression of MFN2 along with miR-195. The mitochondrial division inhibitor Mdivi-1 was purchased from sigma (sigma, USA) and used at 75 µM concentration for 2 h. Cells were fixed post staining using 4% paraformaldehyde and visualized at 63X magnification using a confocal fluorescence microscope (Leica SP8, Germany) for mitochondrial shape and a Nikon Ti-e microscope for quantification of mitochondrial length. The images were processed and mitochondrial branch length of nearly 150 cells was measured using Image-J software.

### RNA isolation and TaqMan assay

The miRNA-195 overexpression and knockdown was analysed using Taqman probes specific to miR-195. The taqMan miRNA assay probes (RT000494 and TM000494) were purchased from applied biosystems. The total RNA was isolated from MCF-7 and MDA-MB-231 cell lines 24 hours post transfections with p195, antimiR-195 and their respective controls. The Trizol reagent (Ambion by Invitrogen, CA, USA) was used as per manufacturer’s instructions to isolate total RNA. The Integrity of RNA samples were checked using a 1% agarose gel in TAE buffer system. Quantification was done using a nanodrop and absorbance measurements at 260 nm, 280 nm and 230 nm. Five hundred nanograms of RNA of each sample was used to synthesize c-DNA using RevertAid H minus first strand cDNA synthesis kit as per manufacturer’s protocol for 18s r-RNA, whereas miR195-specific cDNA was synthesized using the miR-195 specific primer (RT000494) and the TaqMan reverse transcription kit (Applied Biosystems) as per manufacturer’s protocol. The expression of matured miR-195 was estimated using TaqMan miRNA assay probe (TM000494, Applied Biosystems), the 18s r-RNA (Ref-4333760F, Applied Biosystems) was used to normalize the miR-195 expression. Data analysis was done using Pfaffl’s method [53].

### Protein preparation and western blotting

Cells were lysed with RIPA lysis buffer (50 mM Tris–HCl, pH 7.4, 150 mM NaCl, 1% NP40, 0.25% Na-deoxycholate, 1 mM EDTA) containing protease inhibitors (1 μg/ml aprotinin, 1 μg/ml pepstatin, 1 μg/ml leupeptin, 1 mM PMSF,1 mM sodium fluoride and 1 mM sodium orthovanadate) for 30 min on ice. The lysates were centrifuged at 16,000 g for 30 min at 4°C and the supernatant was collected. Protein concentration was estimated by the BCA (Sigma, USA) method. Equal amount of proteins (50–100 μg) were separated on 10%-12% sodium dodecyl sulphate polyacrylamide gel electrophoresis (SDS-PAGE). Separated protein was further transferred to PVDF membrane (Mdi; Advanced Microdevices, India). The membrane was blocked using 5% Bovine serum albumin (BSA) for 1 h at RT and incubated with the respective antibodies in 1% BSA for 16 h at 4⁰C, the blot was washed three times using TBST, and then further incubated with the suitable secondary antibody for 1 h at room temperature. DRP1, OPA1 and MFN2 antibody were purchased from Abcam (Cambridge, UK) and used at 1:1000 concentrations. VDAC1 antibody was purchased from cell signalling and used at 1:1000 concentrations. β-actin and GAPDH were obtained from Sigma (Sigma, USA) and used at 1:5000 concentrations. β–actin and GAPDH were used as protein loading controls to normalize the results. The secondary antibodies were HRP-linked and used at 1:5000 concentration and blots were developed using enhanced chemi-luminiscence (Pierce, Amersham). Integrated density values were calculated using the Image-J software.

### Luciferase reporter assay

The 3′UTR of MFN2 gene was cloned in pSichek2 luciferase vector backbone (Promega, Madison, WI, USA) and the deletion mutant of miR-195 binding site in MFN2 3ʹUTR (deletion in position 1784–1790) was created using quick change-II site directed mutagenesis kit (Agilent technologies, USA). 100 ng of plasmid was used along with the miR-195 treatment in a 12-well cell culture plate. Luciferase activity was measured using the dual luciferase reporter assay system (Promega, Madison, WI, USA) as per manufacturer’s protocol.

### Oxygen consumption rate measurement

For metabolic flux measurement 60,000 to 80,000 cells were seeded in each well of a XF-24 plate, whilst for the plate that was used as a blank (A1, B4, C3, D6) no cells were seeded in the wells. Transfection of miR-195, AntimiR-195 and their respective controls was done using Lipofectamine 2000 (Invitrogen, CA, USA) reagent at 70% to 80% cell confluency. 24 hours post-transfection the cells were washed with XF-24 assay media and incubated in the same buffer for 1 h at 37 ⁰C in a CO_2_-free environment. A pre-hydrated XF-24 cartridge was loaded with inhibitor in their respective chambers. A cartridge with utility plate was kept in XF-24 flux analyser machine and the program was run. After the calibration step, the utility plate was replaced with the cell culture plate and the metabolic flux of the cells was measured. The protein estimation was done using the Bradford method post-experiment to check the equal seeding density in each well of XF-24 flux analyser. The data were normalized per microgram of protein [54].

### Mitochondrial mass measurement

Cells were seeded in 12-well plates and treatment was given once the desired confluency was reached. 50 µM CCCP treatment for 2 h was given as a positive control of mitochondrial mass reduction. Cells were washed post treatment and staining was done using 200 nM mitotracker CMXRos for 15 min at 37 ⁰C. Cells were washed using PBS and harvested. Cells were further acquired using FACS (EasyCyte Guava Technologies).

### Mitochondrial reactive oxygen species measurement

Mitochondrial superoxide was measured using MitosoxRed staining [55]. The cells were seeded in 24 well plate and miR-195 treatment was given at 70% cell confluency. 1 µM rotenone was used as positive control to generate endogenous superoxide radicals. Mitochondrial superoxide was stained using 5 µM MitosoxRed for 20 min at 37 ⁰C. Cells were harvested using 0.5% trypsin and washed with PBS. The cells were acquired using FACS (EasyCyte Guava Technologies).

### Annexin assay

Apoptosis was assessed using Guava Nexin kit (Guava Technologies) that contains annexin V-PE and 7-AAD. The treatments of miR-195, MFN2 plasmid without 3ʹUTR (pMfn2) and Mdivi-1 were given in a 12 well cell culture plate. Cells were treated with 75 µM Mdivi-1 for 2 h before staining. The staining of cells was done as per the manufacturer’s protocol and samples were acquired using FACS (EasyCyte Guava Technologies and BD Accuri C6 Plus).

### Statistical analysis

A two-tailed student t-Test was used for statistical analysis. Mean of a minimum of two or a maximum of four independent experiments ±SE is plotted. P-value less than 0.05, less than 0.01 and less than 0.001 is represented by *, ** and ***, respectively, on plots.
