# Supplementary material for: MiR-195 regulates mitochondrial function by targeting mitofusin-2 in breast cancer cells
Source: RNA Biol. 2019 Apr 25;16(7):918–29. doi: 10.1080/15476286.2019.1600999 (PMC6546347; doi:10.1080/15476286.2019.1600999)
Supplement: Supplemental Material [file krnb-16-07-1600999-s001.zip › Supplementary information/Supplementary Figure 1.docx]

**Supplementary Figure 1. Apoptosis induced by MiR-195 is independent of MFN-2 mediated mitochondrial dysfunction.**

Histogram of Annexin V-PE and 7-AAD binding upon treatment of miR-195, MFN2 along with miR-195, MFN2 and Mdivi-1 in MDAMB-231 cells. **(B)** Plots are representation of relative percentage apoptosis ±SE from n=3 in MDAMB-231, *: *p*<0.05, ***: *p*<0.001.
