# Supplementary figures and images for: MiR-195 regulates mitochondrial function by targeting mitofusin-2 in breast cancer cells
Source: RNA Biol. 2019 Apr 25;16(7):918–29. doi: 10.1080/15476286.2019.1600999 (PMC6546347; doi:10.1080/15476286.2019.1600999)

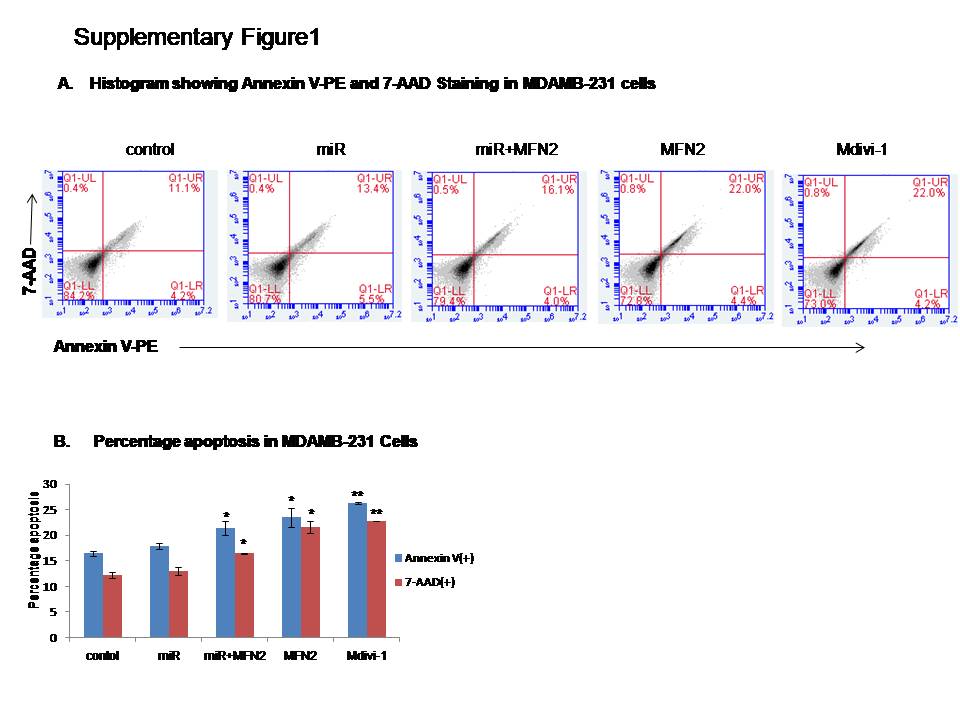

Supplement: Supplemental Material [file krnb-16-07-1600999-s001.zip › Supplementary information/Slide9.JPG]
